# Five Momentous Months

**Published:** 1920-01-31

**Authors:** 


### FIVE MOMENTOUS MONTHS.

### WILL THE VOLUNTARY HOSPITALS BE MADE SECURE?

### *Live Men and Women must rise to the opportunity.*

**Sir Henry Burdett has addressed the following letter to some 300 Hospital Chairmen, Managers and Workers who have already displayed an interest in his scheme for raising a minimum of £25,000,000 for voluntary hospitals in Great Britain.**

Friday's meeting at St. Thomas's Hospital only dealt inferentially with each Hospital's finance, the main theme being the co-operation of the Red Cross organisations and their workers. Agreeing that the Red Cross should occupy the main field at this Meeting I confined my remarks on Friday to the need for money and the relative ease with which it should be obtained by local efforts throughout the country. The justice of this view is strikingly revealed by the latest methods, as demonstrated by the action and brilliant success of the Lord Mayor of Cardiff's effort, which set out to get £150,000. Starting on Thursday, January 15, his efforts brought in half the amount in the first hundred hours, and as we write the whole sum, we understand, is approaching completion.

I venture to point out, for the consideration of all Hospital Chairmen and Managers throughout Great Britain, that it will take time to organise the Red Cross effort, the yield and prospect of which can hardly be known before the late autumn. Meanwhile the course is clear for every Hospital, or a combination of Hospitals in each city and county throughout Great Britain, to set out carefully the claims and needs of the Voluntary Hospitals in its district. Such a statement should give, as Cardiff and other places have done, the full outline of the requirements of the Hospitals, so as to make the appeal inclusive. It is essential to success, in my judgment, that the sum asked for should be large enough to liquidate all outstanding liabilities, and to provide in addition the money needed for extensions, new departments, and other matters which collectively will make the Voluntary Hospitals in the districts where they are placed financially sound, efficiently equipped, and thoroughly up-to-date throughout.

The Lord Mayor of Cardiff held the conviction that what the public wants, and especially shrewd business men, is to be assured and to know that they are asked everywhere to put their money down to support a well-managed and efficient organisation, tireless in overtaking the needs of the population and insistent upon the most perfect administration of the Hospitals for which the money is asked. It is becoming manifestly plain that, although all classes of the population are not insusceptible to sentiment, sentiment is not going to do much good unless it is supported by argument, and argument calls, between now and June 30, for the output of five months' energetic effort by the men and women in every district where there is a voluntary hospital, inviting their personal service to bring the argument home to the people resident therein.

With a view of giving all the help I possibly can to Hospital Workers Everywhere, who have the responsibility of raising large sums of money at this time, I venture to send you this communication, together with a copy of The Hospital of January 24, where on pages 377-9 inclusive you will find the whole position, how to get money successfully, and how to keep it for the Hospital, in succeeding years, and even to increase it, should such a course be necessary. My experience, and I have tested the feeling in many places, is that if once man or woman learns to give personal service in the cause of the sick, like the late King Edward and our present King, time will be found to render such service each year, and year by year, as an act of thankfulness to Almighty God for the blessings of health.

I regret to have to relinquish for a time the somewhat arduous work for which I have been responsible throughout the war and up to the present time. My heart will be with every worker for a Hospital, and with the whole of the people, in every district, where it is decided to secure united action, by all classes, to finance the Voluntary Hospitals. Since my scheme was published in the *Times* it has been warmly received, and already put into action, with results which I hope and believe will encourage the people, everywhere, to follow the noble example of those who have already commenced to organise. They will, I have little doubt, do their utmost to get the money they want by the 30th June next. In my experience results have always been most satisfactory, when they have been produced under a scheme to raise money for Hospitals, within a limited time, for a definite purpose, and a plainly stated amount. Five months is more than ample, when worked on the Cardiff plan, to raise a sum equal to the requirements of any district, however large. I know, however, that there are many ways of raising money, and that in Scotland especially they have a habit of spreading the appeals over a long period. I estimate that three lean years at least have to be faced and provided for, owing to war conditions, so that those who favour the Scottish plan, in preference to my shorter methods, will have ample time to pursue that course, and make it a success, on that basis.

The great thing, whatever plan be adopted, is to get to work at once, and to show by the efforts of the Hospitals and those who already support them, or are willing to support them, that they regard

**Five Momentous Months**—(*continued*).

the Voluntary Hospitals as essential to the well-being of the People. Such evidence will encourage the Red Cross Workers to push ahead and, as I hope and believe, build up an organisation eventually yielding, each year, a total sum for the Hospitals, which with the funds already under their control, or brought to their exchequer through the machinery I have ventured to adumbrate, **will place the Voluntary Hospital System, the finest hospital system in the world, once and for all upon a sound monetary basis.**

May I add that should the rest I am about to take, for a short time, prove as effective as the doctors hope and believe—I have had no holiday since the war began—it will give me very great pleasure to co-operate, all being well, from the beginning of April, with any hospital organisation promoting a scheme on the Cardiff plan in county, municipal, or other districts up and down the country. Meanwhile, my colleagues on the staff of THE HOSPITAL will do all they can to help those engaged in this magnificent work for the voluntary hospitals throughout England, Wales, and Scotland in the preliminary organisation which, to make each appeal effective, must precede the campaign to launch the great appeal and pursue it from the middle of April onwards.

Much thought has convinced me that, so far as London is concerned, the money could be obtained to the required amount in the Metropolis by June 30 next by a system of combination on the part of those hospitals which are in such pressing need of funds to a large amount. **The people of London, like other business communities, require the guarantee of a well-managed and efficient organisation, tireless in overtaking the needs of the population, and insistent upon the most perfect administration of the Hospitals for which the money is asked.** This the hospitals in combination can readily create, and, once created and backed by a statement of what the public wants to know, and the assurance to shrewd business men everywhere that, providing the money is forthcoming, the whole population will speedily be in a position to procure all the needed accommodation and treatment, I make bold to say that, properly led and energetically made known, all the money so urgently required would be speedily forthcoming.

*Note.*—In other large cities, towns, and districts it might be the best plan for all the hospitals serving their population to combine forces, appeals, and workers. The united voice of friendly workers, alert, alive, and whole-heartedly in the work, must win home.

**After all, it must be remembered throughout Great Britain that if the British Red Cross is to be able to do permanently what it so generously aims at doing for the Hospitals, it is up against the Voluntary Hospitals everywhere to shew in the next six months that they will spend and are spending themselves in a great effort to make the Voluntary Hospital System, which is the best in the world, safe for the future and financially.**

I hope everybody who is interested in the Hospitals will take an opportunity of obtaining a copy of THE HOSPITAL for January 24, and reading carefully the article entitled "England, Wales and Scotland Awakening" on pages 377-9. I have made arrangements that any difficulty in obtaining copies of the paper can be overcome by writing to the Manager of The Scientific Press Ltd., 28-29 Southampton Street, Strand, W.C. 2.

If apology be needed to any one for this letter, let it be the fact, that I did not on Friday last make a direct appeal, to cover all the points I have dealt with to-day. The spontaneous offer of the Joint Committee of the British Red Cross Society and Order of St. John is splendid in conception and calculated, I am confident, under sound management—and some very able and tried men will be in responsible charge of the effort—to result, not only in vast benefit to the people of all classes through the Hospital, but in scientific developments which may astonish the world. One thing is certain, Sir Arthur Stanley and the British Red Cross may rest assured, that they will have the whole-hearted co-operation of the ablest, as well as of the humblest workers for the Hospitals, throughout the land.

Yours sincerely and always at your service,

HENRY C. BURDETT,

43 PORCHESTER TERRACE,

LONDON, W. 2,

*January* 28, 1920.

Kindly address all communications to me at THE HOSPITAL BUILDING, 28-29 SOUTHAMPTON STREET, STRAND, LONDON, W.C. 2.

